# Genetic associations with clozapine-induced myocarditis in patients with schizophrenia

**DOI:** 10.1038/s41398-020-0722-0

**Published:** 2020-01-27

**Authors:** Paul Lacaze, Kathlyn J. Ronaldson, Eunice J. Zhang, Ana Alfirevic, Hardik Shah, Leah Newman, Maya Strahl, Melissa Smith, Chad Bousman, Ben Francis, Andrew P. Morris, Trevor Wilson, Fernando Rossello, David Powell, Vivien Vasic, Robert Sebra, John J. McNeil, Munir Pirmohamed

**Affiliations:** 1 Department of Epidemiology and Preventive Medicine, Monash University, Melbourne, VIC Australia; 2 MRC Centre for Drug Safety Science, Wolfson Centre for Personalised Medicine, University of Liverpool, The Royal Liverpool and Broadgreen University Hospitals NHS Trust, and Liverpool Health Partners, Liverpool, UK; 3 Department of Genetics and Genomic Sciences, Icahn Institute for Genomics and Multiscale Biology, Icahn School of Medicine at Mount Sinai, New York, NY USA; 4 Departments of Medical Genetics, Psychiatry, and Physiology & Pharmacology, University of Calgary, Calgary, AB Canada; 5 Department of Biostatistics, University of Liverpool, Liverpool, UK; 6 Division of Musculoskeletal and Dermatological Sciences, University of Manchester, Manchester, UK; 7 Medical Genomics Facility, Hudson Institute of Medical Research, Melbourne, VIC Australia; 8 University of Melbourne Centre for Cancer Research, University of Melbourne, Melbourne, VIC Australia; 9 Bioinformatics Platform, Monash Biomedicine Discovery Institute, Monash University, Melbourne, VIC Australia

**Keywords:** Genetics, Schizophrenia

## Abstract

Clozapine is the most effective antipsychotic drug for schizophrenia, yet it can cause life-threatening adverse drug reactions, including myocarditis. The aim of this study was to determine whether schizophrenia patients with clozapine-induced myocarditis have a genetic predisposition compared with clozapine-tolerant controls. We measured different types of genetic variation, including genome-wide single-nucleotide polymorphisms (SNPs), coding variants that alter protein expression, and variable forms of human leucocyte antigen (HLA) genes, alongside traditional clinical risk factors in 42 cases and 67 controls. We calculated a polygenic risk score (PRS) based on variation at 96 different genetic sites, to estimate the genetic liability to clozapine-induced myocarditis. Our genome-wide association analysis identified four SNPs suggestive of increased myocarditis risk (*P* < 1 × 10^−6^), with odds ratios ranging 5.5–13.7. The SNP with the lowest *P* value was rs74675399 (chr19p13.3, *P* = 1.21 × 10^−7^; OR = 6.36), located in the *GNA15* gene, previously associated with heart failure. The HLA-C*07:01 allele was identified as potentially predisposing to clozapine-induced myocarditis (OR = 2.89, 95% CI: 1.11–7.53), consistent with a previous report of association of the same allele with clozapine-induced agranulocytosis. Another seven HLA alleles, including HLA-B*07:02 (OR = 0.25, 95% CI: 0.05–1.2) were found to be putatively protective. Long-read DNA sequencing provided increased resolution of HLA typing and validated the HLA associations. The PRS explained 66% of liability (*P* value = 9.7 × 10^−5^). Combining clinical and genetic factors together increased the proportion of variability accounted for (*r*^2^ 0.73, *P* = 9.8 × 10^−9^). However, due to the limited sample size, individual genetic associations were not statistically significant after correction for multiple testing. We report novel candidate genetic associations with clozapine-induced myocarditis, which may have potential clinical utility, but larger cohorts are required for replication.

## Introduction

Nearly 50 years after its first commercial use, clozapine continues to be the most effective antipsychotic drug for schizophrenia^1,2^. However, it can cause life-threatening adverse drug reactions, specifically agranulocytosis^3^, gastrointestinal hypomotility^4^ and myocarditis^5^. The risk of agranulocytosis is about 1% and has prompted mandatory blood monitoring protocols in most countries, and restriction to prescription as third-line therapy. Together with concerns about the risk of myocarditis and bowel obstruction, this has reduced the number of patients receiving clozapine, despite its demonstrated superior efficacy.

Myocarditis (inflammation of the heart muscle) occurs in about 3% of patients commencing clozapine, and can be fatal^5^. A protocol has been proposed to monitor myocarditis concurrently with monitoring agranulocytosis in the first 4 weeks following initiation of clozapine^6^. A previous case−control analysis, by our group, designed to identify clinical risk factors for clozapine-induced myocarditis, found the reaction was more common with rapid dose titration, concomitant sodium valproate and increasing age^7^. Despite the investigation of a wide range of host and clinical factors including other concurrent medications, smoking status, alcohol and illicit drug use, body-mass index, and duration of psychiatric illness, the final statistical model accounted for less than 50% of the risk of myocarditis in individuals commencing clozapine. Given the wide range of factors considered, we hypothesized that genetic factors could account for a substantial proportion of the remaining risk.

Clozapine-induced myocarditis is thought to be an immune-mediated reaction, although the mechanism has not been fully elucidated. Pharmacogenetic studies of other immune-mediated adverse drug reactions have identified alleles in the human leucocyte antigen (HLA) region with sufficient predictive value for screening prior to prescribing. Two examples are Stevens−Johnson Syndrome and toxic epidermal necrolysis (SJS/TEN) with carbamazepine in those of South East Asian descent and hypersensitivity with abacavir^8,9^.

However, there are notable clinical differences between the adverse reactions observed with carbamazepine and abacavir, and myocarditis with clozapine. Rechallenge after abacavir hypersensitivity produces a rapid and more severe response than the initial adverse reaction^10^. Rechallenge to carbamazepine after SJS/TEN is strictly contra-indicated. Rechallenge with clozapine after myocarditis may lead to a recurrence; but in around 50% of cases, clozapine has been safely continued long term without discernible adverse cardiac effects^11^. This suggests that there may be several predisposing factors associated with clozapine-induced myocarditis. Indeed, mild cases of myocarditis have been reported, in which clozapine has been continued with doses withheld or temporarily reduced, without any long-term consequences^12^. This would suggest that any genetic predisposition to myocarditis is complex, and that at least for some, risk is modifiable by immunological factors akin to desensitization, or by environmental factors.

The aim of this study was to investigate the contribution of genetic factors—common and rare genetic variants together with HLA gene variants or alleles—to myocarditis risk with clozapine. To our knowledge, this is the first genetic study of clozapine-induced myocarditis.

## Methods

### Sample description

Cases and controls of the cohort have been described previously, along with listing of human research ethics committees from which approvals were obtained^6,7,13^. Briefly, cases met clinical and diagnostic criteria for myocarditis, developing within 45 days of commencing clozapine^13^. Controls took clozapine for at least 45 days with no evidence of myocarditis within this period^13^. Those who met the criteria for case or control were invited via their case manager to provide a blood or saliva sample for genetic analysis. Samples (stored blood or tissue) from deceased individuals were obtained with the consent of the next of kin.

### Genome-wide association analyses

Single-nucleotide polymorphisms (SNPs) were genotyped using the Human OmniExpressExome-8 v1.0 Bead Chip (Illumina, Inc.) in DNA samples from 42 cases, and 67 controls. Sample and SNP quality control (QC) and SNP imputation are described in the Supplementary Materials. A total of 95 samples (33 clozapine-induced myocarditis cases, 62 clozapine-tolerant controls) and 587,647 SNPs passed QC. Phasing and imputation were performed using the Haplotype Reference Consortium (HRC) panel v1.1 2016 ^14^. Post-imputation SNP QC was performed (Supplementary Methods) and a final set of 6,822,414 bi-allelic SNPs remained and were used for association analysis.

Analysis of genotype data was conducted using an additive logistic regression model in SNPTEST v2.5 adjusting for clinical variables reaching statistical significance (*P* < 0.05) and the first four principal components from a genetic relatedness matrix to account for population stratification. The conventional genome-wide association study (GWAS) threshold of *P* < 5 × 10^−8^ was regarded as genome-wide significant (Bonferroni-corrected for 1 million SNPs). Since this threshold is conservative for small sample sizes, SNPs with *P* *<* 1 × 10^−5^ were considered suggestive of genome-wide significance.

### Polygenic risk score analysis

A polygenic risk score (PRS) calculates the aggregate weighted contribution of multiple low-effect genetic loci (SNPs) on a given trait or disease outcome. We calculated a PRS for clozapine-induced myocarditis using a sub-set of SNPs meeting a more stringent QC threshold, using the PRSice-2 software^15^ (Supplementary Materials). We adjusted for clinical variables and the first four principal components to control for population stratification. The *R*^2^ value was calculated to estimate the proportion of explained variance in myocarditis status.

### Rare coding variant analyses

Of the SNPs assayed on the Illumina Human OmniExpressExome-8 v1.0 Bead Chip, 244,331 were coding (exonic) variants affecting protein expression. These were used to undertake a limited analysis of rare coding variants contributing to clozapine-induced myocarditis risk (see Supplementary Methods).

### HLA analyses

HLA typing was conducted using short- and long-read methods. Short-read HLA calls were made using the Omixon Target HLA Typing software (v1.8.1), following hybridization capture (SureSelect MHC, Agilent) and DNA sequencing (2 × 150 bp paired end, Illumina HiSeq). Long-read, high-resolution HLA typing was performed using long-range PCR amplification and long-read single molecule real-time DNA sequencing (Pacific Biosciences RSII). For this approach, input DNA was amplified using HLA Class I and II NGSgoAmpX primers (GenDX) and purified to targeted amplicon sizes (~3–4 kb for HLA-A, -B and -C). After PCR products were normalized and pooled, they were subject to size selection, template preparation, barcode multiplexing (eight libraries per pool) and SMRT sequencing using magnetic bead selection protocol. Long-read data were processed using the Long Amplicon Analysis pipeline v2 (Pacific Biosciences). FASTQ files were imported into the NGSengine v2.7.0 software (GenDX) for HLA allele calling, SNP identification and individual base calling.

Short-read DNA sequencing enabled HLA typing at standard (4-digit) resolution. This detected 29 HLA loci and 4 non-HLA loci across the major histocompatibility complex (MHC) (Table S1). Loci with call rates <98% were excluded (*n* = 7). Of the remaining 26 loci, 313 alleles were detected (Table S2). Monomorphic alleles (*n* = 25) and alleles with counts of <1 (*n* = 86) were excluded. To minimize co-linearity, only SNPs with correlation coefficient *r*^2^ < 0.8 were included. A final number of 127 HLA and 32 non-HLA alleles in 33 cases and 62 controls were included for downstream analyses. HLA alleles were grouped into supertypes (Table S3) according to published classifications^16,17^. The frequency of HLA haplotype was explored in publicly available Caucasoid populations using the ‘haplotype frequency search’ option on the allele frequency net database.

To evaluate the effect of each individual HLA allele, HLA supertype and HLA haplotype on clozapine-induced myocarditis, logistic regression analysis was undertaken using PLINK software^18^, assuming a dominant mode of inheritance, with adjustment for clinical variables with *P* < 0.05. Correction for multiple testing was undertaken using Benjamini−Hochberg false discovery rate (FDR)^19^ in the genetics package of R (v3.3.1).

Long-read HLA sequencing was performed to improve accuracy and resolution of the short-read HLA sequencing results, to resolve ambiguities and to generate high-resolution (eight-digit) HLA calls. Long-read sequencing detected 11 HLA loci (*HLA-A, B, C, DRB1, DRB3, DRB4, DRB5, DQA1, DQB1, DPA1*, and *DPB1*). HLA loci with >90% call rate (HLA-A, B, and C) were included in the concordance check with short-read HLA sequencing data.

### Clinical variables and statistical analyses

Statistical associations of clinical variables (sex, smoking, alcohol abuse, illicit drug use, valproate use, chronic disease, age, BMI, and cumulative clozapine dose) with clozapine-induced myocarditis were evaluated using either the Pearson’s chi-square test or ANOVA (see Supplementary Methods). Clinical covariates found to be significantly associated with clozapine-induced myocarditis were combined with genetic factors using regression models, to calculate the proportion of variability accounted for.

## Results

Table 1 provides a summary of the case−control cohort, clinical variables and genomic measures, as well as numbers included in each analysis. Exposure to potentially interacting medication via cytochrome P450 metabolism was minimal among cases and controls (*n* = 0–4; Table S4).

**Table 1:** Cohort description and numbers.

	Cases	Controls
Number	42	67
Sample type		
Fresh blood/saliva/stored blood/tissue	20/11/6/5	61/6/0/0
Age at clozapine initiation		
Mean age (years) ± SD	37 ± 12	35 ± 11
Reported ethnicity		
European/other/unknown	36/2/4	57/7/3
Outcome fatal	24% (10/42)	—
Sex (% male)	71% (30/42)	81% (54/67)
Clinical factors		
Smoking—Y/N/Unknown	29/7/6	49/18/0
Alcohol abuse—Y/N/Unknown	10/25/7	9/46/12
Illicit drug use—Y/N/Unknown	8/27/7	12/46/9
Valproate use—Y/N/Unknown	12/26/4	9/58/0
BMI kg/m^2^ ± SD	31.7 ± 6.8 (*n* = 30)	27.8 ± 6.5 (*n* = 54)
Cumulative clozapine dose—days 1–9		
Mean (mg) ± SD	751 ± 222 (*n* = 35)	691 ± 289 (*n* = 62)
Genetic analyses—QC pass rate^a^		
1. Genome-wide SNP association	79% (33/42)	93% (62/67)
2. Rare variants/gene-level analysis	71% (30/42)	81% (54/67)
3. Targeted short-read HLA typing	93% (33/42)	96% (62/67)
4. Long-read HLA typing/phasing	71% (30/42)	90% (60/67)

^a^For reasons for exclusion, see Supplementary methods.

### Genome-wide single-nucleotide polymorphism (SNP) association

**Fig. 1:**
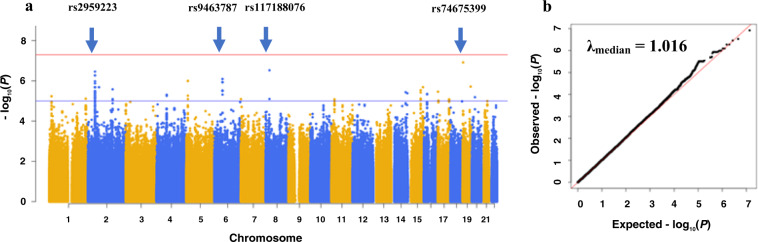
Association of genome-wide genetic variants in clozapine-induced myocarditis cases compared with clozapine-tolerant controls. The first four principal components and valproate use were included as covariates. **a** Manhattan plot showing individual −log_10_ *P* values plotted against their genomic position by chromosome. The red line represents the threshold of genome-wide significance (*P* = 5 × 10^−8^). The blue line marks the threshold for suggestive association (*P* = 1 × 10^−5^). Blue arrows indicate SNPs with *P* *<* 1 × 10^−6^. **b** Quantile−quantile (Q−Q) plot of data shown in Manhattan plot.

**Fig. 2:**
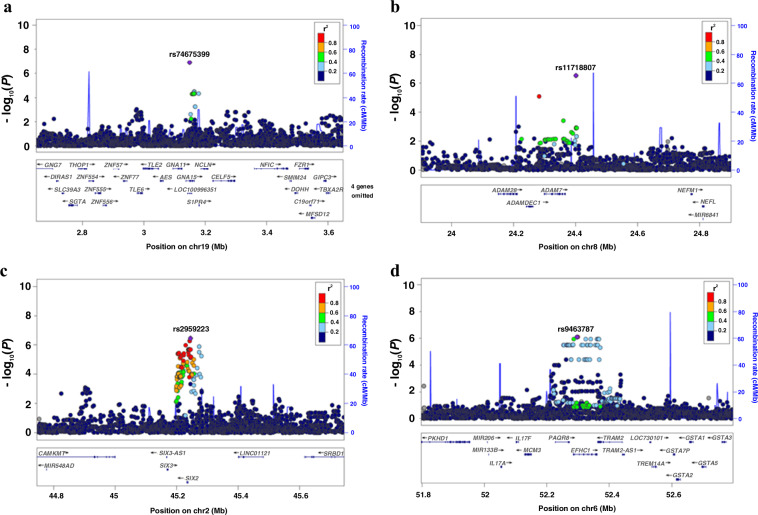
Regional plots of SNP regions with associations *P* < 1 × 10^−6^. Plots are shown for *GNA15* on chromosome 19p13.3 (**a**), *ADAM7* on chromosome 8p21.2 (**b**), *SIX2-SIX3* on chromosome 2p21 (**c**), and *EFHC1* on chromosome 6p12.2 (**d**). Variants with the lowest *P* value at each locus are identified by rsID (purple diamond). Each coloured dot represents a SNP *in our results*, with the colour scale reflecting the degree of LD (*r*^2^) with the most significant variant. Physical positions are based on assembly GRCh37.p13 of the human genome. SNPs with missing LD information are shown in grey. Regional plots were created using LocusZoom.

Our analysis found no SNPs reaching genome-wide significance between cases and controls (Fig. 1). However, SNPs with suggestive genome-wide significance were observed in several regions. In particular, there were four SNPs located on chromosomes 2p21, 6p12.2, 8p21.2, and 19p13.3 (Table 2, Fig. 2) with *P* *<* 1 × 10^−6^. The frequencies of these four variants were at least 20% higher in cases compared to controls.

**Table 2:** SNPs associated with clozapine-induced myocarditis with *P* < 1 × 10^−6^.

rsID	Chr	Base pair	Gene(s)	Location relative to gene	Risk allele/ other allele	Genotype frequency						Risk allele frequency		OR	95% CI	*P v*alue	Impute quality
						AA AB BB											
						Cases			Controls			Cases	Controls				
rs2959223	2	45245165	*SIX3, SIX2*	Intergenic	A/G	0.38	0.52	0.10	0.08	0.44	0.48	0.64	0.30	5.55	1.44–21.26	3.50E–07	0.99
rs9463787	6	52296583	*EFHC1*	Intronic	G/A	0.21	0.55	0.24	0.02	0.23	0.76	0.48	0.13	7.67	2.63–22.36	8.08E–07	0.99
rs117188076	8	24401398	*ADAM7*	Intergenic	T/C	0.01	0.44	0.55	0.00	0.06	0.94	0.23	0.03	13.74	3.68–51.22	2.98E–07	0.89
rs74675399	19	3148869	*GNA15*	Intronic	A/G	0.04	0.50	0.47	0.00	0.14	0.86	0.28	0.07	6.36	2.28–17.8	1.21E–07	0.88

The most significant variant associated with clozapine-induced myocarditis at each locus is listed.

*Chr* chromosome, *BP* base pair positions based on assembly GRCh37.p13 of the human genome, *OR* odds ratio that reflects the comparison between heterozygotes versus wild-type homozygotes, *CI* confidence interval.

The SNP with the lowest *P* value was rs74675399 located on chromosome 19p13.3 (*P* = 1.21 × 10^−7^; OR = 6.36; Fig. 2a), within intron 2 of the *GNA15* gene. This gene encodes the G-protein subunit alpha-15 and has previously been reported in genome-wide association with heart failure^20^. The second lowest *P* value was associated with an intergenic SNP, rs117188076 (*P* = 2.98 × 10^−7^; OR = 13.74; Fig. 2b), located on chromosome 8p21.2. This variant is located ~31 kb from the 3′ end of the disintegrin and metalloproteinase domain-containing protein 7 gene (*ADAM7*). The third was also an intergenic SNP, rs2959223 (*P* = 3.5 × 10^−7^; OR = 5.55; Fig. 2c), positioned ~8.6 kb from the 3′ end of transcription factor *SIX2* that encodes for homeobox protein SIX2 on chromosome 2p21. The fourth was an intronic *EFHC1* SNP, rs9463787 (*P* *=* 8.08 × 10^−7^; OR = 7.67; Fig. 2d) on chromosome 6p12.2. *EFHC1* encodes the EF-hand-containing calcium binding protein, which plays a role in calcium homoeostasis.

### Polygenic risk score (PRS)

**Fig. 3:**
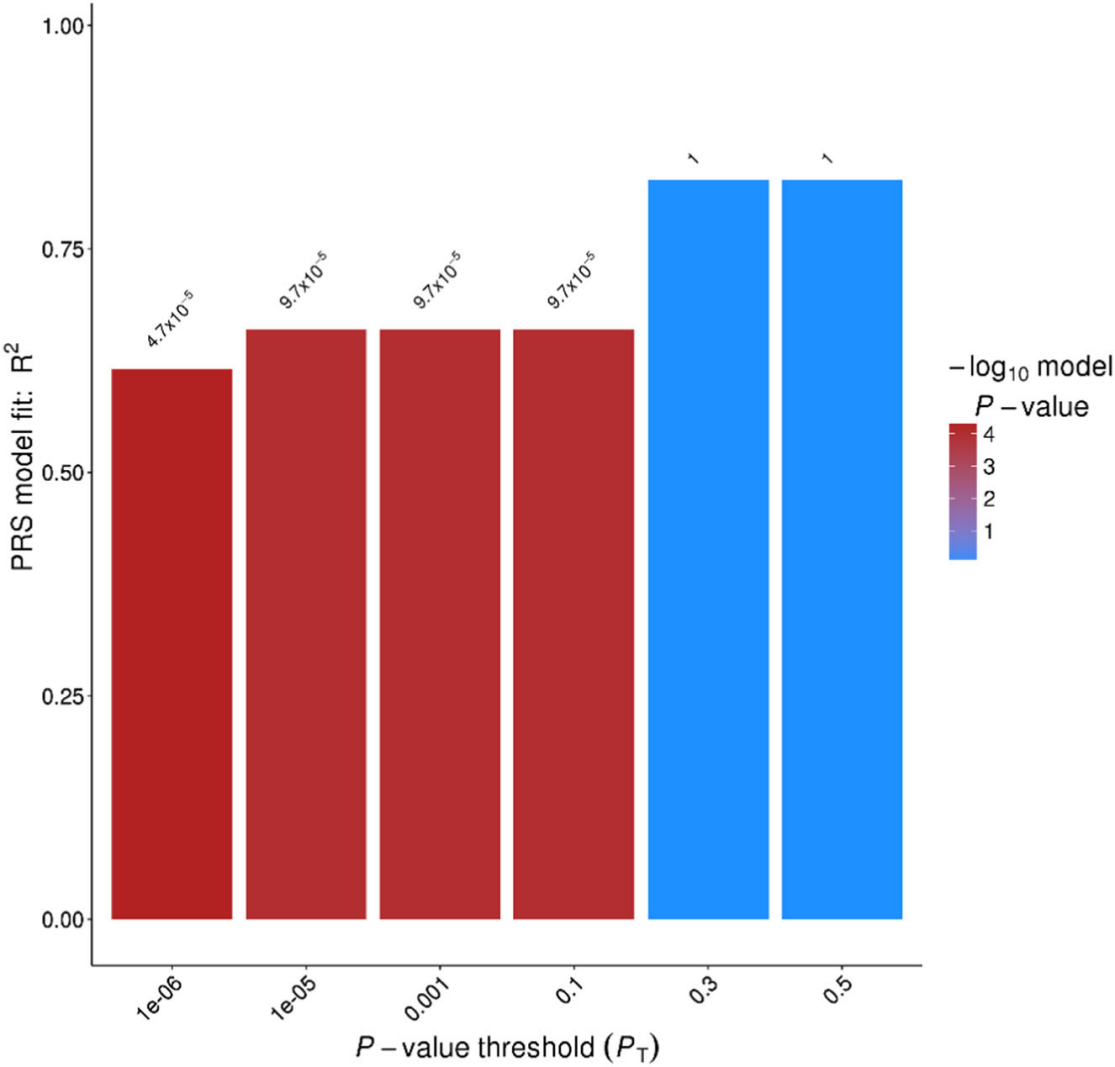
Polygenic risk score analysis using GWAS genetic variants. Risk scores of pruned SNPs at each predetermined significance threshold were calculated using aggregated sum of weighted risk allele effect sizes. The proportion of variance (*R*^2^) explained by the aggregate risk score is shown on the *y*-axis.

PRS was calculated using SNP effect sizes (odds ratio) from the GWAS analysis. Ninety-six SNPs with *P* < 1 × 10^−5^ explained 66% of variance in clozapine-induced myocarditis (PRS *P* value = 9.7 × 10^−5^) (Fig. 3).

### Exonic variants/gene-based association

A total of 226,140 exonic variants in protein-coding gene regions genotyped across 30 clozapine-induced myocarditis cases, and 54 clozapine-tolerant controls passed quality control for gene-based analysis. No significant association was found between the exonic markers and clozapine-induced myocarditis with regard to detectable disruption in gene function (Fig. S1).

### HLA association

The short-read DNA sequencing enables HLA typing to two-digit resolution. This identified nine HLA gene variant (allele) associations with clozapine-induced myocarditis (Table 3), prior to false discovery rate (FDR) correction to address type I errors. HLA class I allele HLA-C*07:01 (OR = 2.89, 95% CI: 1.11–7.53; *P* = 0.03) and HLA class II allele HLA-DMB*01:01 (OR = 3.22, 95% CI: 1.158.97; *P* = 0.026) were associated with increased odds for myocarditis.

**Table 3:** Associations of HLA and non-HLA alleles with clozapine-induced myocarditis showing *P* < 0.05 prior to FDR. Valproate use was included in the analyses as covariate.

Allele/supertype	Cases		Controls		OR (95% CI)	*P*^a^
	Carrier	Non-carrier	Carrier	Non-carrier		
HLA Class I						
HLA-B*07:02	0.10	0.90	0.32	0.68	0.26 (0.07–0.98)	0.047
HLA-C*07:01	**0.55**	**0.45**	**0.34**	**0.66**	**2.89** (**1.11**–**7.53)**	**0.030**^**a**^
HLA-C*07:02	0.10	0.90	0.34	0.66	0.25 (0.07–0.92)	0.037
HLA Class II						
HLA-DMB*01:01	**0.76**	**0.24**	**0.52**	**0.48**	**3.22** (**1.15**–**8.97)**	**0.026**^**a**^
HLA-DQA1*01:02	0.17	0.83	0.45	0.55	0.23 (0.074–0.71)	0.011
HLA-DQB1*06:02	0.10	0.90	0.35	0.65	0.17 (0.045–0.69)	0.012
HLA-DRA*01:01	0.34	0.66	0.68	0.32	0.27 (0.11–0.70)	0.007
HLA-DRB1*15:01	0.10	0.90	0.35	0.65	0.17 (0.045–0.69)	0.012
Non-HLA						
MICB*004:01	0.21	0.79	0.48	0.52	0.27 (0.09–0.77)	0.015
HLA supertype						
HLA Class II DR1^b^	0.17	0.83	0.39	0.61	0.28 (0.09–0.89)	0.031

^a^*P* values did not remain significant after FDR.

^b^The HLA-DRB1 supertype consists of DRB1*15:01, *15:02, *15:03, *15:04, *16:01, and *16:02.

Lines in bold text indicate increased risk of myocarditis.

The other seven HLA alleles were all associated with decreased frequency of myocarditis (ORs < 1). Two of these were HLA class I alleles (HLA-B*07:02 and HLA-C*07:02) and four were HLA class II alleles (HLA-DQA1*01:02, HLA-DQB1*06:02, HLA-DRA*01:01 and HLA-DRB1*15:01). One was a non-HLA allele, MICB*004:01. In addition to allele associations, one HLA supertype, DR1, exhibited a significant association with decreased frequency of clozapine-induced myocarditis (OR < 1; Table 3). However, none of the allelic and supertype associations remained significant after FDR.

Using the allele frequency net database^21^, HLA-B*07:02, DQB1*06:02 and DRB1*15:01 were found to be in the same haplotype group. Among 44 records focusing on the Caucasoid ethnic origin across seven populations, consisting of a total of 1,250,628 individuals, the estimated occurrence of the HLA-B*07:02, DQB1*06:02 and DRB1*15:01 haplotypes ranges between 0.01 and 11%. In our cohort of clozapine-induced myocarditis cases and controls, the carrier frequency of these three alleles together was 9.1% and 22.6%, respectively (OR = 0.25, 95% CI: 0.05–1.2; *P* = 0.083). This suggests that differences observed in HLA-B*07:02, DQB1*06:02 and DRB1*15:01 frequencies between cases and controls in our study are not likely to be due to confounding differences in population structure.

### Long-read HLA typing

Long-read DNA sequencing can span highly variable HLA gene regions, to enable typing at higher resolution, to four-digits. Long-read sequencing was used to validate and further resolve HLA calls made by short-read sequencing in clozapine-induced myocarditis cases and controls. Among the 96 samples that underwent long-read HLA DNA sequencing, 90 had short-read HLA sequencing data available, of which 86 had successful long-read HLA-B PCR results (Table S5).

All HLA-B*07:02 calls based on short-read data were identified as HLA-B*07:02:01:01 by long-read analysis. Eighty samples had successful long-read HLA-C results. All short-read calls for HLA-C*07:01 and *07:02 were concordant with long-read typing. Carriers of the loci previously associated with clozapine-induced agranulocytosis^22^, HLA-C*07:01 (*n* = 31), were identified as having HLA-C*07:01:01:01 (*n* = 30) and HLA-C*07:01:01:04 (*n* = 1; control). The HLA-C*07:02 carriers (*n* = 31) were identified as having HLA-C*07:02:01:03 (*n* = 30) and HLA-C*07:02:01:01 (*n* = 1; case). Discordance between HLA typing calls from short- and long-read DNA sequencing was observed across five samples (Table S6), but this did not alter the statistical findings of the short-read HLA analysis.

### Regression analysis for myocarditis with clozapine

The univariate associations of clinical variables with clozapine-induced myocarditis are summarized in Table S7. We found that the concomitant use of valproate with clozapine contributes 6.8% to the risk of myocarditis (OR = 3.1, 95% CI: 1.1–8.9; *P* = 0.03) (Table 4). The addition of HLA and non-HLA alleles increased the *r*^2^ by approximately 24%. When the top four GWAS SNPs were included, a further 42% increase in *r*^2^ was observed. When clinical and genetic factors were combined, they explained a higher proportion of risk (72.6%) compared to clinical factors alone.

**Table 4:** Contribution of clinical and genetic variables to clozapine-induced myocarditis.

Variable	Nagelkerke’s *r*^2^	*P*
Clinical only (valproate use)	0.068	0.029
Clinical + HLA^a^	0.307	0.021
Clinical + HLA + GWAS^b^	**0.726**	**9.838E–09**

^a^Included HLA-B*07:02, HLA-C*07:01, HLA-C*07:02, HLA-DMB*01:01, HLA-DQA1*01:02, HLADQB1*06:02, HLA-DRA*01:01, HLA-DRB1*15:01, and MICB*004:01. HLA-DRB1*15:01 was excluded from the model due to redundancy.

^b^Included GWAS top four associations rs2959223, rs9463787, rs117188076, rs74675399. Bold text indicates statistical signifcance *P* < 0.001.

## Discussion

This study provides the first evidence, to our knowledge, that genetic factors contribute to the risk of myocarditis with clozapine. Although the genetic associations identified did not reach the conventional statistical threshold used in human genetics, and require replication in larger studies, they provide a novel set of candidate genetic loci for this serious adverse drug reaction, and may be of potential clinical utility.

A strength of our study is the comprehensive genetic analyses undertaken using a range of technologies used to measure different types of genetic variation, including genome-wide SNP variation, HLA variation and polygenic risk. Our results have prompted collaborative efforts to increase sample sizes to improve statistical power to detect difference, through an international clozapine-induced myocarditis consortium^23^.

Several previous genetic studies have been conducted on clozapine-induced agranulocytosis^24^. These suggest agranulocytosis with clozapine may be a heritable trait, with a complex genetic architecture where numerous genetic loci contribute, including the HLA genes^24^, in combination with clinical factors. Further, a recent genome-wide association study (GWAS) of neutropenia in patients taking clozapine identified an association with *HLA-DQB*1^25^. Together, these previous studies suggest a small, but significant role of genetic factors contributing to the risk of clozapine-induced agranulocytosis and neutropenia, but no single polymorphism with high predictive value or current clinical utility. Similarly, the results of our study suggest that the risk of myocarditis with clozapine is underpinned by a complex genetic architecture.

Following either myocarditis or agranulocytosis/neutropenia, the outcome of rechallenge with clozapine may be favourable^11,26^. In addition, clozapine has been safely continued in mild cases of myocarditis^12^. These observations suggest that the pathogenesis may not always be a typical immune-mediated reaction, but one where a complex combination of genetic and clinical factors lead to predisposition, and modification of one or more of these, or a process akin to desensitization, may modulate risk.

From a biological standpoint, we identified genetic risk loci that provide clues to the underlying aetiology of myocarditis with clozapine. These loci include four novel SNPs that approached genome-wide significance, even in our small sample size. While we found no associations above the standard statistical threshold for genome-wide SNP significance (*P* < 5 × 10^−8^), there are instances of SNPs discovered below this threshold being biologically meaningful, and reaching genome-wide significance subsequently, after larger sample sizes are available^27^. We therefore find it reasonable to interpret the biological consequences and potential clinical utility of the genetic variation we have identified, albeit with due caution.

The locus with the lowest associated *P* value was located within intron 2 of the *GNA15* gene and is associated with six-fold increase in susceptibility to clozapine-induced myocarditis. This locus has previously been associated with risk of incident heart failure in adults of European ancestry^20^. The *GNA15* gene encodes a Ga/q protein that plays a role in a variety of signalling pathways important for heart function^28^. *GNA15* sits in a genomic region (19p13.3) where microdeletions have been linked to congenital heart disease^29^. The implicated variant (rs74675399) sits 132 base pairs upstream from an enhancer region and 1.7 kilobases downstream of a promoter flanking region, suggesting it may act as a proxy for functional genomic elements. Further, clozapine has high binding affinities to receptors that *GNA15* is functionally coupled to, such as 5-HT2 serotonergic, muscarinic (M1, M3, M4), alpha-1 adrenergic, and histamine H1 receptors^30^. *GNA15* is also involved in lymphocyte and neutrophil activation after pro-inflammatory cytokine treatment^31,32^. Even with the caveats of limited sample size and conservative type 1 error threshold, this provides a promising candidate locus with biological plausibility, related to the clinical phenotype observed.

With regards to HLA associations, statistical thresholds and FDR correction also apply to reduce the possibility of type I error. We found potential novel HLA associations with clozapine-induced myocarditis, including the HLA class I allele HLA-C*07:01 (OR = 2.89, 95% CI: 1.11–7.53; *P* = 0.03), previously associated with clozapine-induced agranulocytosis^22^. This result suggested an overlapping role of HLA-C*07:01 in the risk of different clinically significant clozapine-induced adverse drug reactions. Interestingly, HLA-C*07:01 has also been associated with the response to peg-interferon alfa-2b and ribavirin therapy in hepatitis C^33^. When we typed C*07:01 patients using high-resolution, long-read HLA sequencing, we found 30/31 carriers shared the HLA-C*07:01:01:01 sub-type.

We identified seven HLA alleles associated with the decreased frequency (risk) of clozapine-induced myocarditis (ORs < 1), including the HLA-B*07:02 allele (OR = 0.26, 95% CI: 0.07–0.98; *P* = 0.047). HLA B*07:02 has been previously associated, by our group, with putative protective function against carbamazepine hypersensitivity (Stevens−Johnson syndrome) in Caucasian patients^34^. High-resolution typing showed that all HLA-B*07:02 carriers were HLA-B*07:02:01:01 sub-types. Of all patients with HLA-B calls by long-read sequencing, the HLA-B*07:02:01:01 allele was present in 18 of 55 controls (33%) but only 4 of 33 (12%) cases (*P* = 0.0375).

Together, our results indicate contributions from multiple genetic loci and HLA alleles to the risk of myocarditis with clozapine, suggesting a complex and polygenic architecture^35^. To explore this further, we generated a polygenic risk score using 96 SNPs (*P* < 1 × 10^−5^). This explained 66% of the liability to clozapine-induced myocarditis (*P* = 9.7 × 10^−5^). This was a high proportion of variability for an exploratory PRS model, and provides encouraging evidence that use of genetic information may benefit risk management of clozapine-induced myocarditis.

In our previous case−control study examining clinical risk factors for clozapine-induced myocarditis (105 cases, 296 controls), we found that risk of myocarditis in individuals commencing clozapine was increased by rapid dose titration, greater patient age and concomitant sodium valproate use^7^. In the present study, we confirmed the association with valproate use (*r*^2^ 0.07, *P* = 0.03), and found that combining clinical and genetic factors together substantially increased the proportion of variability accounted for (*r*^2^ 0.73, *P* = 9.8 × 10^−9^). This provided further evidence that the addition of genetic information to clinical risk models for clozapine-induced myocarditis may be of potential future clinical utility in risk management and prevention. However, the genetic associations identified require validation in larger replication studies.

There could also be yet undiscovered genetic factors contributing to myocarditis risk not detected by our current methods. These include rare, high-effect size genetic variants detectable by whole-genome or whole-exome sequencing, large structural variants, or functional haplotypes in genes relevant to clozapine metabolism (e.g. *CYP1A2)*.

### Limitations

The small sample size of the cohort is the most notable limitation of this study. We spent a number of years and considerable resources recruiting even this small number of patients, encountering a range of challenges. These included the low population frequency of schizophrenia (prevalence ~1/100), the restrictions on clozapine prescribing, the reluctance of psychiatrists to prescribe clozapine due to its adverse effects, and the rarity of the drug reaction amongst patients commencing clozapine (estimated 3/100 patients treated). The stringency of clinical criteria used to define cases also contributed^13^. The sample set was further narrowed by the mental illness of some potential participants who were not able to be contacted about contributing a sample, or had cognitive barriers to participation. Poor DNA quality also contributed to failures for some of the genomic assays used, further reducing statistical power. In addition, with regards to measuring rare exonic variants, our analysis was restricted to only a small sub-set of coding variants included on the genotyping array used.

### Conclusion

We report possible novel genetic associations with clozapine-induced myocarditis, some of which overlap with related adverse drug reactions. Our results provide candidate mechanisms by which clozapine could induce myocardial inflammation and damage, and identify candidate genetic loci that may, in aggregate, be of future clinical utility for preventing a serious and sometimes fatal adverse drug reaction. Our results warrant further investigations for this important clinical phenotype, and validation in larger studies.

## Supplementary information

Supplementary Materials (Data and Methods)
