# Supplementary material for: Genetic associations with clozapine-induced myocarditis in patients with schizophrenia
Source: Transl Psychiatry. 2020 Jan 27;10:37. doi: 10.1038/s41398-020-0722-0 (PMC7026069; doi:10.1038/s41398-020-0722-0)
Supplement: Supplementary file 1 — Supplementary Materials (Data and Methods) [file 41398_2020_722_MOESM1_ESM.docx]

**Supplementary Materials (Data & Methods)**

**Methods**

*Genome-wide genotyping*

Genotype calling was performed using the GenomeStudio Software 2011.1. Quality control prior to imputation included removal of samples with reported gender discordance, genotyping success rate ≤ 95%, or heterozygosity rate >3 standard deviations from the mean. To confirm the ethnic origin of samples, our genotypic data were anchored by genotype data from the HapMap 3 populations and multidimensional scaling (MDS) was undertaken using PLINK (20). To eliminate systematic bias due to population stratification, samples of non-European ancestry were removed. Cryptic relatedness was also assessed by calculating identity by descent (IBD) using PLINK (20) and no kinship was detected in our cohort of patients.

SNPs were excluded if the call rate was ≤95%, the minor allele frequency (MAF) was <1%, a significant difference (*P* < 1 x 10^-5^) in call rate was observed between cases and controls, or deviated from Hardy-Weinberg equilibrium *P* < 1 x 10^-6^ in controls. In addition, the Haplotype Reference Consortium (HRC) preparation and checking tool ([well.ox.ac.uk/~wrayner/tools/](http://www.well.ox.ac.uk/~wrayner/tools/)) was used. After quality control, genotype data were available for a total of 587,647 SNP markers across 33 clozapine-induced myocarditis cases and 62 clozapine tolerant controls. Phasing and imputation were performed with the HRC reference panel v1.1 2016 on the Michigan Imputation Server (13) using Eagle v2.3 and Minimac 3 respectively. A total of 41,583,724 SNPs were imputed. After QC (Minimac Rsq threshold > 0.3, MAF > 1%, HWE > 0.000001), 6,822,414 bi-allelic SNPs remained for analysis. The extent of genomic inflation was assessed by calculating the genomic control inflation factor, λ (the ratio of the median observed chi-square statistic with one degree of freedom to that expected from the same distribution) (15) in R (v3.3.1).

*Polygenic risk score analysis*

SNPs with *P*<0.5 from our GWAS analysis were pruned based on *r*^2^ < 0.2 where *r*^2^ was a measure of LD between the observed and expected frequencies of haplotypes. Pruned SNPs were ranked by their strength of association with clozapine-induced myocarditis and were restricted to predetermined significance thresholds (*P* < 1x10^-6^, 1x10^-5^, 0.001, 0.1, 0.3, 0.5). A polygenic risk score (PRS) was calculated, adjusting for clinical variables and the first four principal components to control for population stratification. The R^2^ value, was calculated to estimate the proportion of explained variance in myocarditis status.

*Rare exomic variant calling*

The Illumina Human OmniExpressExome-8 v1.0 Bead Chip contains 244,331 exome variants. Given that it is recommended to use at least 1000 samples for zCalling (16), sample and SNP QC for exonic variants were performed together with 2529 external samples from the University of Liverpool, followed by joint calling using the zCall algorithm. After joint zCalling, a secondary sample and SNP QC was performed. Samples with gender mismatch, call rate <90%, non-European ethnicity, heterozygosity rate > 3 standard deviations from the mean, and cryptic relatedness were excluded. SNPs with call rate < 95%, Hardy-Weinberg equilibrium *P* < 1 x 10^-4^, cluster separation score < 0.4, and GenTrain score < 0.6 were removed. After zCalling, a secondary QC was performed and exclusions included samples with call rate < 99%, heterozygosity rate > 7 standard deviations from the mean and SNPs with call rate < 99%. A total of 226,140 exome variants across 30 clozapine-induced myocarditis cases and 54 clozapine-tolerant controls remained for downstream analysis.

The functional effects of exonic variants were previously predicted using five in silico algorithms (PolyPhen-2 HumDiv and HumVar, SIFT, LRT, and MutationTaster). Based on these functional annotations, loss of function and nonsynonymous variants with MAF<5% were filtered into two groups for gene-based analyses: 1) ‘Broad’ - any variants predicted to be deleterious by at least one algorithm; and 2) ‘Strict’ - variants predicted to be deleterious by all five algorithms. The SKAT-O test within the Efficient and Parallelizable Association Container Toolbox (EPACTS) was used to perform the broad and strict gene-based tests. The first four principal components were included as covariates. Statistical significance for gene-based analyses was defined as P<2.5x10-6 (Bonferroni-corrected for ~20,000 genes).

*HLA typing by hybridization capture and short read DNA sequencing*

SureSelect MHC Capture design (#0404051/61, protocol G7530-90000 Version 1.5) was used following Manual v6, 31.05.2011 (Agilent) to conduct target DNA capture by hybridisation (32,762 probes designed to Hg19). Libraries were generated without indexes or sequencing adaptors, according to modified methods for DNA capture with insert size of mean 500bp (6 cycles of amplification, 500ng DNA input per capture/library). Indexes were added during post capture amplification (12 Cycles). Samples were pooled for sequencing according to DNA quantity (Qubit, Life Technologies), using a size range of 350-600bp, including adaptors, sequencing primers and indexes. Pools were run on 2% agarose gel and size isolated, re-quantitated by Bioanalyzer (Agilent) and Qubit (Life Technologies), with 12pM used for cluster generation. Sequencing was conducted using the HiSeq platform (2 x 150bp paired end) using protocol 15035788 Rev A, Oct 2012. (Illumina). PhiX spike-in was included as a control, error rate 0.49±0.12%. Sequencing data were subsequently processed using the Omixon Target HLA Typing software v1.8.1.

*Target enrichment of HLA regions using long-range PCR*

Target enrichment for HLA Class I and Class II regions was performed by PCR amplification of genomic DNA, quantified using the Qubit HS dsDNA kit (Thermofisher Scientific). For all amplicons except DQB1, 200 ng of sample was added to the Qiagen LongRange PCR mix of 0.4μl LongRange Enzyme, 2.5μl LongRange Buffer, 1.25μl dNTP mix and water for a final volume of 24μl. For the DQB1 amplicon, 200 ng of sample was added to the Qiagen LongRange PCR mix of 5.0μl Q Solution, 0.8μl LongRange Enzyme, 2.5μl LongRange Buffer, 1.25μl dNTP mix and water for a final volume of 24μl. HLA Class I and II GenDX NGSgo-AmpX kit primers were used for amplification and resuspended in 108μl of nuclease free water (GenDX). 1.0μl of HLA Class I and Class II primer was then added per reaction for a final reaction volume of 25μl. Initial denaturation per reaction was 95°C for 3 minutes, then for a total of 35 cycles; denaturation at 95°C for 15 seconds, primer annealing at 65°C for 30 seconds and elongation at 68°C for 5 minutes. After the 35^th^ cycle, reactions were brought through a final elongation at 68°C for 10 minutes with a cooling step at 15°C overnight. After amplification, PCR products were quantified using the Qubit HS dsDNA kit (Thermofisher) and the Agilent DNA 12000 Bioanalyzer kit to identify correct amplicon sizes based on the GenDX NGSgo-AmpX Kit approximations. For HLA-A, -B and -C products, sizes were 3-4kb and for DRB1 and DRB3 sizes were 3.7-4kb. DRB4 and DPB1 contained 2 expected products; DRB4 at 400bp and 1.5kb, and DPB1 at 5.0kb and 5.7kb respectively. DRB5 products were 4kb in size, DQA1 were 5.5-5.8kb DQB1 amplicons were 3.7-4kb and DPA1 amplicons were 4.7kb in size.) After amplification, PCR products were purified with a bead wash of 0.6X volume of AMPure PB Beads (Pacific Biosciences, part no. 100-265-900) and eluted in 30μl of elution buffer (Zymo Research).

*Single-molecule long read library preparation, DNA sequencing and HLA typing*

Purified PCR products were normalized to equal concentrations and pooled with 10 amplicons per pool (exluding DRB4), per sample and quantified using the Qubit HS dsDNA kit (Thermofisher) and the Agilent DNA 12000 Bioanalyzer kit. 500ng total of each pooled sample was then brought through the Pacific Biosciences Amplicon Template Preparation and Sequencing protocol for SMRTbell library preparation, using the SMRTbell template Prep Kit 1.0 (part no. 100-222-300) and 0.6X AMPure PB bead size selection. SMRTbell libraries for DRB4 amplicons were prepped separately in order to adequately sequence the 400bp exon 2, using a 1.0X Ampure PB bead size selection. Each library was individually barcoded using SMRTbell Barcoded Adapters (part no. 100-465-900) and multiplexed with 8 libraries per pool.

Sequencing primer annealing was performed using the recommended 20:1 primer:template ratio, whereas P6 polymerase binding was performed at a modified polymerase:template ratio of 3:1 for DRB4 amplicon SMRTbells and 10:1 ratio for all other SMRTbells. HLA SMRTbell libraries were immobilized onto SMRT cells at a starting concentration of 150 pM on chip for DRB4 amplicons and 70 pM on chip for all others. Loading titrations were performed to achieve optimal sequencing conditions for particular samples as necessary. SMRT sequencing was performed on the RS II using the C3 magnetic bead and diffusion run (DRB4 SMRTbell libraries) loading and 360-minute movies. Long Amplicon Analysis pipeline v2 (LAA2) reads generated .FASTQ files which were imported into the NGSengine v2.7.0 (GenDX) software for allele calling, SNP identification and individual base calling. A filter of mapability of >98% removed poor quality assignments. No further filters for read-depth were used.

*Clinical and combinatorial model analyses*

Statistical analyses were conducted using SPSS v24. Univariate associations of clinical variables (sex, smoking, alcohol abuse, illicit drug use, valproate use, chronic disease, age, BMI, and cumulative clozapine dose) with clozapine-induced myocarditis were evaluated using either the Pearson’s chi-square test or ANOVA. Binary logistic regression models were built and compared using the likelihood ratio test (LRT), and the proportion of variability for each regression model was calculated using Nagelkerke’s r2 statistic. The baseline “clinical” model included significant clinical variables (P < 0.05) from the univariate analyses. Two “genetic” models were fitted. The first included clinical covariates found significant in the univariate analyses and genetic factors from the HLA analyses. The second genetic model was the same but also included genetic variants from the GWAS analyses.

**Results**

**Table S1: List of HLA and non-HLA loci detected by short-read sequencing**

|  | | |
| --- | --- | --- |
| **Locus** | **Call Rate (%)** | **Comments** |
|  |  |  |
| HLA Class I |  |  |
| HLA-A | 100 | Include |
| HLA-B | 100 | Include |
| HLA-C | 100 | Include |
| HLA-E | 100 | Include |
| HLA-F | 100 | Include |
| HLA-G | 100 | Include |
|  |  |  |
| HLA Class I (Pseudogene) | |  |
| HLA-H | 100 | Include |
| HLA-J | 100 | Include |
| HLA-K | 100 | Include |
| HLA-L | 100 | Include |
| HLA-V | 100 | Include |
|  |  |  |
| HLA Class II |  |  |
| HLA-DMA | 100 | Include |
| HLA-DMB | 100 | Include |
| HLA-DOA | 100 | Include |
| HLA-DOB | 100 | Include |
| HLA-DPA1 | 100 | Include |
| HLA-DPB1 | 100 | Include |
| HLA-DQA1 | 100 | Include |
| HLA-DQB1 | 100 | Include |
| HLA-DRA | 100 | Include |
| HLA-DRB1 | 100 | Include |
|  |  |  |
| HLA Class II (Pseudogene) | |  |
| HLA-DRB2 | 56.36 | Exclude |
| HLA-DRB3 | 27.27 | Exclude |
| HLA-DRB4 | 60.00 | Exclude |
| HLA-DRB5 | 30.91 | Exclude |
| HLA-DRB6 | 48.18 | Exclude |
| HLA-DRB7 | 56.36 | Exclude |
| HLA-DRB8 | 56.36 | Exclude |
| HLA-DRB9 | 100.00 | Include |
|  |  |  |
| Non-HLA |  |  |
| MICA | 100 | Include |
| MICB | 100 | Include |
| TAP1 | 100 | Include |
| TAP2 | 100 | Include |

| **Table S2: List of alleles detected on each HLA and non-HLA locus.** | | | | | | | |
| --- | --- | --- | --- | --- | --- | --- | --- |
| **Locus** | | **Allele Name** | | **Four Digit Allele Name** | | **Comments** | |
|  | |  | |  | |  | |
| HLA Class I | |  | |  | |  | |
| HLA-A* | | 01:01:01 | | 01:01 | |  | |
| HLA-A* | | 02:01:01 | | 02:01 | |  | |
| HLA-A* | | 02:02 | | 02:02 | | Allele count ≤ 1 in either cases or controls | |
| HLA-A* | | 02:05:01 | | 02:05 | |  | |
| HLA-A* | | 02:17:02 | | 02:17 | | Allele count ≤ 1 in either cases or controls | |
| HLA-A* | | 02:197 | | 02:197 | | Monomorphic | |
| HLA-A* | | 03:01:01 | | 03:01 | |  | |
| HLA-A* | | 03:01:07 | | 03:01 | |  | |
| HLA-A* | | 03:02:01 | | 03:02 | | Allele count ≤ 1 in either cases or controls | |
| HLA-A* | | 11:01:01 | | 11:01 | |  | |
| HLA-A* | | 23:01:01 | | 23:01 | | Allele count ≤ 1 in either cases or controls | |
| HLA-A* | | 24:02:01 | | 24:02 | |  | |
| HLA-A* | | 25:01:01 | | 25:01 | |  | |
| HLA-A* | | 29:01:01 | | 29:01 | | Allele count ≤ 1 in either cases or controls | |
| HLA-A* | | 29:02:01 | | 29:02 | |  | |
| HLA-A* | | 29:05 | | 29:05 | | Allele count ≤ 1 in either cases or controls | |
| HLA-A* | | 30:01:01 | | 30:01 | | Allele count ≤ 1 in either cases or controls | |
| HLA-A* | | 30:26 | | 30:26 | | Monomorphic | |
| HLA-A* | | 31:01:02 | | 31:01 | |  | |
| HLA-A* | | 32:01:01 | | 32:01 | | Allele count ≤ 1 in either cases or controls | |
| HLA-A* | | 33:01:01 | | 33:01 | | Allele count ≤ 1 in either cases or controls | |
| HLA-A* | | 33:03:01 | | 33:03 | | Allele count ≤ 1 in either cases or controls | |
| HLA-A* | | 34:01:01 | | 34:01 | | Monomorphic | |
| HLA-A* | | 68:01:01 | | 68:01 | |  | |
| HLA-A* | | 68:01:02 | | 68:01 | |  | |
| HLA-A* | | 68:02:01 | | 68:02 | | Allele count ≤ 1 in either cases or controls | |
| HLA-A* | | 69:01 | | 69:01 | | Allele count ≤ 1 in either cases or controls | |
|  | |  | |  | |  | |
| HLA-B* | | 07:02:01 | | 07:02 | |  | |
| HLA-B* | | 07:05:01 | | 07:05 | | Allele count ≤ 1 in either cases or controls | |
| HLA-B* | | 07:06 | | 07:06 | | Allele count ≤ 1 in either cases or controls | |
| HLA-B* | | 07:35 | | 07:35 | | Monomorphic | |
| HLA-B* | | 08:01:01 | | 08:01 | |  | |
| HLA-B* | | 13:02:01 | | 13:02 | | Allele count ≤ 1 in either cases or controls | |
| HLA-B* | | 14:01:01 | | 14:01 | |  | |
| HLA-B* | | 14:02:01 | | 14:02 | | Allele count ≤ 1 in either cases or controls | |
| HLA-B* | | 15:01:01 | | 15:01 | |  | |
| HLA-B* | | 15:02:01 | | 15:02 | | Monomorphic | |
| HLA-B* | | 15:07:01 | | 15:07 | | Allele count ≤ 1 in either cases or controls | |
| HLA-B* | | 15:21 | | 15:21 | | Monomorphic | |
| HLA-B* | | 15:27:01 | | 15:27 | | Monomorphic | |
| HLA-B* | | 18:01:01 | | 18:01 | |  | |
| HLA-B* | | 27:02:01 | | 27:02 | | Allele count ≤ 1 in either cases or controls | |
| HLA-B* | | 27:05:02 | | 27:05 | |  | |
| HLA-B* | | 35:01:01 | | 35:01 | |  | |
| HLA-B* | | 35:02:01 | | 35:02 | | Allele count ≤ 1 in either cases or controls | |
| HLA-B* | | 35:03:01 | | 35:03 | | Allele count ≤ 1 in either cases or controls | |
| HLA-B* | | 35:08:01 | | 35:08 | |  | |
| HLA-B* | | 37:01:01 | | 37:01 | | Allele count ≤ 1 in either cases or controls | |
| HLA-B* | | 38:01:01 | | 38:01 | | Allele count ≤ 1 in either cases or controls | |
| HLA-B* | | 38:02:01 | | 38:02 | |  | |
| HLA-B* | | 38:06 | | 38:06 | | Monomorphic | |
| HLA-B* | | 39:01:01 | | 39:01 | | Allele count ≤ 1 in either cases or controls | |
| HLA-B* | | 39:06:02 | | 39:06 | | Allele count ≤ 1 in either cases or controls | |
| HLA-B* | | 39:24:01 | | 39:24 | |  | |
| HLA-B* | | 40:01:02 | | 40:01 | |  | |
| HLA-B* | | 40:02:01 | | 40:02 | | Allele count ≤ 1 in either cases or controls | |
| HLA-B* | | 41:01 | | 41:01 | |  | |
| HLA-B* | | 44:02:01 | | 44:02 | |  | |
| HLA-B* | | 44:03:01 | | 44:03 | |  | |
| HLA-B* | | 45:01 | | 45:01 | | Allele count ≤ 1 in either cases or controls | |
| HLA-B* | | 49:01:01 | | 49:01 | |  | |
| HLA-B* | | 50:01:01 | | 50:01 | |  | |
| HLA-B* | | 51:01:01 | | 51:01 | |  | |
| HLA-B* | | 55:01:01 | | 55:01 | | Allele count ≤ 1 in either cases or controls | |
| HLA-B* | | 57:01:01 | | 57:01 | |  | |
| HLA-B* | | 58:01:01 | | 58:01 | |  | |
|  | |  | |  | |  | |
| HLA-C* | | 01:02:01 | | 01:02 | |  | |
| HLA-C* | | 02:02:02 | | 02:02 | |  | |
| HLA-C* | | 03:02:02 | | 03:02 | |  | |
| HLA-C* | | 03:03:01 | | 03:03 | |  | |
| HLA-C* | | 03:04:01 | | 03:04 | |  | |
| HLA-C* | | 04:01:01 | | 04:01 | |  | |
| HLA-C* | | 04:01:43 | | 04:01 | |  | |
| HLA-C* | | 04:03:01 | | 04:03 | | Monomorphic | |
| HLA-C* | | 04:09N | | 04:09N | |  | |
| HLA-C* | | 04:59Q | | 04:59Q | | Allele count ≤ 1 in either cases or controls | |
| HLA-C* | | 05:01:01 | | 05:01 | |  | |
| HLA-C* | | 06:02:01 | | 06:02 | |  | |
| HLA-C* | | 06:116N | | 06:116N | | Monomorphic | |
| HLA-C* | | 07:01:01 | | 07:01 | |  | |
| HLA-C* | | 07:01:02 | | 07:01 | |  | |
| HLA-C* | | 07:02:01 | | 07:02 | |  | |
| HLA-C* | | 07:02:15 | | 07:02 | |  | |
| HLA-C* | | 07:04:01 | | 07:04 | | Allele count ≤ 1 in either cases or controls | |
| HLA-C* | | 07:18 | | 07:18 | | Allele count ≤ 1 in either cases or controls | |
| HLA-C* | | 08:01:01 | | 08:01 | | Monomorphic | |
| HLA-C* | | 08:02:01 | | 08:02 | | Allele count ≤ 1 in either cases or controls | |
| HLA-C* | | 12:03:01 | | 12:03 | |  | |
| HLA-C* | | 14:02:01 | | 14:02 | |  | |
| HLA-C* | | 15:02:01 | | 15:02 | |  | |
| HLA-C* | | 15:05:02 | | 15:05 | | Allele count ≤ 1 in either cases or controls | |
| HLA-C* | | 16:01:01 | | 16:01 | |  | |
| HLA-C* | | 16:02:01 | | 16:02 | | Allele count ≤ 1 in either cases or controls | |
| HLA-C* | | 16:04:01 | | 16:04 | | Allele count ≤ 1 in either cases or controls | |
| HLA-C* | | 17:01:01 | | 17:01 | | Allele count ≤ 1 in either cases or controls | |
|  | |  | |  | |  | |
| HLA-E* | | 01:01:01 | | 01:01 | |  | |
| HLA-E* | | 01:01:02 | | 01:01 | |  | |
| HLA-E* | | 01:03:01 | | 01:03 | |  | |
| HLA-E* | | 01:03:02 | | 01:03 | |  | |
| HLA-E* | | 01:03:05 | | 01:03 | |  | |
| HLA-E* | | 01:06 | | 01:06 | |  | |
|  | |  | |  | |  | |
| HLA-F* | | 01:01:01 | | 01:01 | |  | |
| HLA-F* | | 01:01:02 | | 01:01 | |  | |
| HLA-F* | | 01:01:03 | | 01:01 | |  | |
| HLA-F* | | 01:03:01 | | 01:03 | |  | |
| HLA-F* | | 01:04 | | 01:04 | |  | |
|  | |  | |  | |  | |
| HLA-G* | | 01:01:01 | | 01:01 | |  | |
| HLA-G* | | 01:01:02 | | 01:01 | |  | |
| HLA-G* | | 01:01:03 | | 01:01 | |  | |
| HLA-G* | | 01:01:04 | | 01:01 | |  | |
| HLA-G* | | 01:01:08 | | 01:01 | |  | |
| HLA-G* | | 01:01:12 | | 01:01 | |  | |
| HLA-G* | | 01:01:14 | | 01:01 | |  | |
| HLA-G* | | 01:01:17 | | 01:01 | |  | |
| HLA-G* | | 01:01:18 | | 01:01 | |  | |
| HLA-G* | | 01:03:01 | | 01:03 | |  | |
| HLA-G* | | 01:04:01 | | 01:04 | |  | |
| HLA-G* | | 01:04:03 | | 01:04 | |  | |
| HLA-G* | | 01:04:04 | | 01:04 | |  | |
| HLA-G* | | 01:05N | | 01:05N | | Allele count ≤ 1 in either cases or controls | |
| HLA-G* | | 01:06 | | 01:06 | |  | |
| HLA-G* | | 01:08 | | 01:08 | | Allele count ≤ 1 in either cases or controls | |
|  | |  | |  | |  | |
| HLA Class I (Pseudogene) | | | |  | |  | |
| HLA-H* | | 01:01:01 | | 01:01 | |  | |
| HLA-H* | | 01:02 | | 01:02 | |  | |
| HLA-H* | | 02:01:01 | | 02:01 | |  | |
| HLA-H* | | 02:02 | | 02:02 | |  | |
| HLA-H* | | 02:03 | | 02:03 | | Allele count ≤ 1 in either cases or controls | |
| HLA-H* | | 02:04 | | 02:04 | |  | |
| HLA-H* | | 02:05 | | 02:05 | |  | |
| HLA-H* | | 02:06 | | 02:06 | | Allele count ≤ 1 in either cases or controls | |
|  | |  | |  | |  | |
| HLA-J* | | 01:01:01 | | 01:01 | | Monomorphic | |
|  | |  | |  | |  | |
| HLA-K* | | 01:01:01 | | 01:01 | |  | |
| HLA-K* | | 01:02 | | 01:02 | |  | |
| HLA-K* | | 01:03 | | 01:03 | |  | |
|  | |  | |  | |  | |
| HLA-L* | | 01:01:01 | | 01:01 | |  | |
| HLA-L* | | 01:01:02 | | 01:01 | |  | |
| HLA-L* | | 01:02 | | 01:02 | |  | |
|  | |  | |  | |  | |
| HLA-V* | | 01:01:01 | | 01:01 | | Monomorphic | |
|  | |  | |  | |  | |
| HLA Class II | |  | |  | |  | |
| HLA-DMA* | | 01:01:01 | | 01:01 | |  | |
| HLA-DMA* | | 01:02 | | 01:02 | |  | |
|  | |  | |  | |  | |
| HLA-DMB* | | 01:01:01 | | 01:01 | |  | |
| HLA-DMB* | | 01:02 | | 01:02 | |  | |
| HLA-DMB* | | 01:03:01 | | 01:03 | |  | |
| HLA-DMB* | | 01:04 | | 01:04 | |  | |
| HLA-DMB* | | 01:05 | | 01:05 | | Monomorphic | |
| HLA-DMB* | | 01:07 | | 01:07 | |  | |
|  | |  | |  | |  | |
| HLA-DOA* | | 01:01:01 | | 01:01 | | Allele count ≤ 1 in either cases or controls | |
| HLA-DOA* | | 01:01:02 | | 01:01 | | Allele count ≤ 1 in either cases or controls | |
| HLA-DOA* | | 01:01:03 | | 01:01 | | Allele count ≤ 1 in either cases or controls | |
| HLA-DOA* | | 01:01:04 | | 01:01 | | Allele count ≤ 1 in either cases or controls | |
| HLA-DOA* | | 01:01:05 | | 01:01 | | Allele count ≤ 1 in either cases or controls | |
| HLA-DOA* | | 01:01:06 | | 01:01 | | Allele count ≤ 1 in either cases or controls | |
| HLA-DOA* | | 01:02 | | 01:02 | | Allele count ≤ 1 in either cases or controls | |
| HLA-DOA* | | 01:04N | | 01:04N | | Monomorphic | |
|  | |  | |  | |  | |
| HLA-DOB* | | 01:01:01 | | 01:01 | |  | |
| HLA-DOB* | | 01:01:03 | | 01:01 | |  | |
| HLA-DOB* | | 01:02:01 | | 01:02 | |  | |
| HLA-DOB* | | 01:03 | | 01:03 | |  | |
| HLA-DOB* | | 01:04:01 | | 01:04 | |  | |
| HLA-DOB* | | 01:05 | | 01:05 | |  | |
|  | |  | |  | |  | |
| HLA-DPA1* | | 01:03:01 | | 01:03 | |  | |
| HLA-DPA1* | | 01:04 | | 01:04 | | Allele count ≤ 1 in either cases or controls | |
| HLA-DPA1* | | 02:01:01 | | 02:01 | |  | |
| HLA-DPA1* | | 02:01:02 | | 02:01 | |  | |
| HLA-DPA1* | | 02:02:02 | | 02:02 | |  | |
|  | |  | |  | |  | |
| HLA-DPB1* | | 01:01:01 | | 01:01 | |  | |
| HLA-DPB1* | | 02:01:02 | | 02:01 | |  | |
| HLA-DPB1* | | 02:02 | | 02:02 | | Allele count ≤ 1 in either cases or controls | |
| HLA-DPB1* | | 03:01:01 | | 03:01 | |  | |
| HLA-DPB1* | | 04:01:01 | | 04:01 | |  | |
| HLA-DPB1* | | 04:02:01 | | 04:02 | |  | |
| HLA-DPB1* | | 05:01:01 | | 05:01 | |  | |
| HLA-DPB1* | | 06:01 | | 06:01 | | Allele count ≤ 1 in either cases or controls | |
| HLA-DPB1* | | 10:01 | | 10:01 | |  | |
| HLA-DPB1* | | 11:01:01 | | 11:01 | |  | |
| HLA-DPB1* | | 13:01:01 | | 13:01 | | Allele count ≤ 1 in either cases or controls | |
| HLA-DPB1* | | 14:01 | | 14:01 | | Monomorphic | |
| HLA-DPB1* | | 15:01 | | 15:01 | | Allele count ≤ 1 in either cases or controls | |
| HLA-DPB1* | | 16:01 | | 16:01 | | Allele count ≤ 1 in either cases or controls | |
| HLA-DPB1* | | 17:01 | | 17:01 | | Allele count ≤ 1 in either cases or controls | |
| HLA-DPB1* | | 20:01:01 | | 20:01 | | Allele count ≤ 1 in either cases or controls | |
| HLA-DPB1* | | 23:01 | | 23:01 | |  | |
| HLA-DPB1* | | 34:01 | | 34:01 | | Allele count ≤ 1 in either cases or controls | |
| HLA-DPB1* | | 64:01N | | 64:01N | | Allele count ≤ 1 in either cases or controls | |
| HLA-DPB1* | | 104:01 | | 104:01 | | Allele count ≤ 1 in either cases or controls | |
| HLA-DPB1* | | 105:01 | | 105:01 | |  | |
| HLA-DPB1* | | 126:01 | | 126:01 | |  | |
|  | |  | |  | |  | |
| HLA-DQA1* | | 01:01:01 | | 01:01 | |  | |
| HLA-DQA1* | | 01:01:02 | | 01:01 | |  | |
| HLA-DQA1* | | 01:02:01 | | 01:02 | |  | |
| HLA-DQA1* | | 01:02:02 | | 01:02 | |  | |
| HLA-DQA1* | | 01:03:01 | | 01:03 | |  | |
| HLA-DQA1* | | 01:04:01 | | 01:04 | |  | |
| HLA-DQA1* | | 01:05 | | 01:05 | | Allele count ≤ 1 in either cases or controls | |
| HLA-DQA1* | | 01:12 | | 01:12 | | Allele count ≤ 1 in either cases or controls | |
| HLA-DQA1* | | 02:01 | | 02:01 | |  | |
| HLA-DQA1* | | 03:01:01 | | 03:01 | |  | |
| HLA-DQA1* | | 03:02 | | 03:02 | |  | |
| HLA-DQA1* | | 03:03:01 | | 03:03 | |  | |
| HLA-DQA1* | | 04:01:01 | | 04:01 | |  | |
| HLA-DQA1* | | 04:01:02 | | 04:01 | |  | |
| HLA-DQA1* | | 05:01:01 | | 05:01 | |  | |
| HLA-DQA1* | | 05:03 | | 05:03 | | Allele count ≤ 1 in either cases or controls | |
| HLA-DQA1* | | 05:05:01 | | 05:05 | |  | |
| HLA-DQA1* | | 06:01:01 | | 06:01 | | Monomorphic | |
|  | |  | |  | |  | |
| HLA-DQB1* | | 02:01:01 | | 02:01 | |  | |
| HLA-DQB1* | | 02:01:08 | | 02:01 | |  | |
| HLA-DQB1* | | 02:02:01 | | 02:02 | |  | |
| HLA-DQB1* | | 02:06 | | 02:06 | | Monomorphic | |
| HLA-DQB1* | | 03:01:01 | | 03:01 | |  | |
| HLA-DQB1* | | 03:02:01 | | 03:02 | |  | |
| HLA-DQB1* | | 03:03:02 | | 03:03 | |  | |
| HLA-DQB1* | | 03:05:01 | | 03:05 | | Allele count ≤ 1 in either cases or controls | |
| HLA-DQB1* | | 03:19 | | 03:19 | | Allele count ≤ 1 in either cases or controls | |
| HLA-DQB1* | | 04:01:01 | | 04:01 | | Monomorphic | |
| HLA-DQB1* | | 04:02:01 | | 04:02 | |  | |
| HLA-DQB1* | | 05:01:01 | | 05:01 | |  | |
| HLA-DQB1* | | 05:02:01 | | 05:02 | |  | |
| HLA-DQB1* | | 05:03:01 | | 05:03 | |  | |
| HLA-DQB1* | | 06:01:01 | | 06:01 | | Monomorphic | |
| HLA-DQB1* | | 06:02:01 | | 06:02 | |  | |
| HLA-DQB1* | | 06:03:01 | | 06:03 | |  | |
| HLA-DQB1* | | 06:04:01 | | 06:04 | | Allele count ≤ 1 in either cases or controls | |
| HLA-DQB1* | | 06:05:01 | | 06:05 | | Monomorphic | |
| HLA-DQB1* | | 06:09:01 | | 06:09 | | Allele count ≤ 1 in either cases or controls | |
|  | |  | |  | |  | |
| HLA-DRA* | | 01:01:01 | | 01:01 | |  | |
| HLA-DRA* | | 01:01:02 | | 01:01 | |  | |
| HLA-DRA* | | 01:02:02 | | 01:02 | |  | |
| HLA-DRA* | | 01:02:03 | | 01:02 | |  | |
|  | |  | |  | |  | |
| HLA-DRB1* | | 01:01:01 | | 01:01 | |  | |
| HLA-DRB1* | | 01:02:01 | | 01:02 | | Allele count ≤ 1 in either cases or controls | |
| HLA-DRB1* | | 01:03 | | 01:03 | | Allele count ≤ 1 in either cases or controls | |
| HLA-DRB1* | | 03:01:01 | | 03:01 | |  | |
| HLA-DRB1* | | 03:01:11 | | 03:01 | |  | |
| HLA-DRB1* | | 04:01:01 | | 04:01 | |  | |
| HLA-DRB1* | | 04:02:01 | | 04:02 | | Allele count ≤ 1 in either cases or controls | |
| HLA-DRB1* | | 04:03:01 | | 04:03 | |  | |
| HLA-DRB1* | | 04:04:01 | | 04:04 | |  | |
| HLA-DRB1* | | 04:05:01 | | 04:05 | |  | |
| HLA-DRB1* | | 04:06:01 | | 04:06 | | Monomorphic | |
| HLA-DRB1* | | 04:07:01 | | 04:07 | | Allele count ≤ 1 in either cases or controls | |
| HLA-DRB1* | | 04:08:01 | | 04:08 | | Allele count ≤ 1 in either cases or controls | |
| HLA-DRB1* | | 07:01:01 | | 07:01 | |  | |
| HLA-DRB1* | | 08:01:03 | | 08:01 | |  | |
| HLA-DRB1* | | 08:03:02 | | 08:03 | | Allele count ≤ 1 in either cases or controls | |
| HLA-DRB1* | | 09:01:02 | | 09:01 | |  | |
| HLA-DRB1* | | 10:01:01 | | 10:01 | | Allele count ≤ 1 in either cases or controls | |
| HLA-DRB1* | | 11:01:01 | | 11:01 | |  | |
| HLA-DRB1* | | 11:02:01 | | 11:02 | | Allele count ≤ 1 in either cases or controls | |
| HLA-DRB1* | | 11:03 | | 11:03 | | Allele count ≤ 1 in either cases or controls | |
| HLA-DRB1* | | 11:04:01 | | 11:04 | |  | |
| HLA-DRB1* | | 12:01:01 | | 12:01 | | Allele count ≤ 1 in either cases or controls | |
| HLA-DRB1* | | 12:02:01 | | 12:02 | | Monomorphic | |
| HLA-DRB1* | | 13:01:01 | | 13:01 | | Allele count ≤ 1 in either cases or controls | |
| HLA-DRB1* | | 13:02:01 | | 13:02 | | Allele count ≤ 1 in either cases or controls | |
| HLA-DRB1* | | 13:03:01 | | 13:03 | | Allele count ≤ 1 in either cases or controls | |
| HLA-DRB1* | | 14:01:01 | | 14:01 | | Allele count ≤ 1 in either cases or controls | |
| HLA-DRB1* | | 14:05:01 | | 14:05 | | Monomorphic | |
| HLA-DRB1* | | 14:54:01 | | 14:54 | |  | |
| HLA-DRB1* | | 15:01:01 | | 15:01 | |  | |
| HLA-DRB1* | | 15:02:01 | | 15:02 | | Monomorphic | |
| HLA-DRB1* | | 16:01:01 | | 16:01 | | Allele count ≤ 1 in either cases or controls | |
| HLA-DRB1* | | 16:02:01 | | 16:02 | | Allele count ≤ 1 in either cases or controls | |
|  | |  | |  | |  | |
| HLA Class II (Pseudogene) | | | |  | |  | |
| HLA-DRB9* | | 01:01 | | 01:01 | | Monomorphic | |
|  | |  | |  | |  | |
| Non-HLA | |  | |  | |  | |
| MICA* | | 001 | | 001 | | Allele count ≤ 1 in either cases or controls | |
| MICA* | | 002:01 | | 002:01 | |  | |
| MICA* | | 004 | | 004 | |  | |
| MICA* | | 006 | | 006 | | Allele count ≤ 1 in either cases or controls | |
| MICA* | | 007:01 | | 007:01 | |  | |
| MICA* | | 008:01:01 | | 008:01 | |  | |
| MICA* | | 008:02 | | 008:02 | | Allele count ≤ 1 in either cases or controls | |
| MICA* | | 008:04 | | 008:04 | |  | |
| MICA* | | 009:01 | | 009:01 | |  | |
| MICA* | | 009:02 | | 009:02 | |  | |
| MICA* | | 010:01 | | 010:01 | |  | |
| MICA* | | 011 | | 011 | | Allele count ≤ 1 in either cases or controls | |
| MICA* | | 012:01 | | 012:01 | | Allele count ≤ 1 in either cases or controls | |
| MICA* | | 016 | | 016 | |  | |
| MICA* | | 017 | | 017 | |  | |
| MICA* | | 018:01 | | 018:01 | |  | |
| MICA* | | 019 | | 019 | | Allele count ≤ 1 in either cases or controls | |
| MICA* | | 027 | | 027 | |  | |
| MICA* | | 047 | | 047 | |  | |
| MICA* | | 049 | | 049 | |  | |
| MICA* | | 052 | | 052 | | Allele count ≤ 1 in either cases or controls | |
| MICA* | | 068 | | 068 | |  | |
|  | |  | |  | |  | |
| MICB* | | 002:01:01 | | 002:01 | |  | |
| MICB* | | 003 | | 003 | |  | |
| MICB* | | 004:01:01 | | 004:01 | |  | |
| MICB* | | 005:01 | | 005:01 | |  | |
| MICB* | | 005:02:04 | | 005:02 | |  | |
| MICB* | | 005:03 | | 005:03 | | Allele count ≤ 1 in either cases or controls | |
| MICB* | | 005:06 | | 005:06 | | Allele count ≤ 1 in either cases or controls | |
| MICB* | | 008 | | 008 | |  | |
| MICB* | | 010 | | 010 | |  | |
| MICB* | | 012 | | 012 | | Monomorphic | |
| MICB* | | 014 | | 014 | |  | |
| MICB* | | 024 | | 024 | | Allele count ≤ 1 in either cases or controls | |
| MICB* | | 028 | | 028 | |  | |
|  | |  | |  | |  | |
| TAP1* | | 01:01:01 | | 01:01 | |  | |
| TAP1* | | 02:01:01 | | 02:01 | |  | |
| TAP1* | | 02:01:02 | | 02:01 | |  | |
| TAP1* | | 03:01 | | 03:01 | |  | |
| TAP1* | | 04:01 | | 04:01 | | Allele count ≤ 1 in either cases or controls | |
| TAP1* | | 05:01 | | 05:01 | |  | |
|  | |  | |  | |  | |
| TAP2* | | 01:01:01 | | 01:01 | |  | |
| TAP2* | | 01:01:02 | | 01:01 | |  | |
| TAP2* | | 01:01:03 | | 01:01 | |  | |
| TAP2* | | 01:02 | | 01:02 | |  | |
| TAP2* | | 01:03 | | 01:03 | | Allele count ≤ 1 in either cases or controls | |
| TAP2* | | 01:04 | | 01:04 | |  | |
| TAP2* | | 02:01:01 | | 02:01 | |  | |
| TAP2* | | 02:01:02 | | 02:01 | |  | |

# **Table S3: HLA Supertypes: Grouped according to previously published classification (18, 19).**

| **HLA Locus** | **Supertype** | **HLA Allele** |
| --- | --- | --- |
| HLA Class I |  |  |
| HLA-A | A01 | 01:01, 26:01, 26:02, 30:02, 30:04, 32:01; 01:03, 26:07, 26:08, 36:01, 25:01, 32:08, 80:01 |
| HLA-A | A01_A03 | 30:01, 68:07 |
| HLA-A | A01_A24 | 29:02, 29:01 |
| HLA-A | A02 | 02:01, 02:02, 02:03, 02:05, 02:06, 02:07, 02:17, 68:02, 69:01; 02:11, 02:13, 02:16, 02:20, 02:24, 02:27, 02:30, 02:38, 02:77; 02:08 |
| HLA-A | A03 | 03:01, 11:01, 31:01, 33:01, 33:03, 66:01, 68:01, 74:01; 03:02, 11:02, 11:04, 31:09, 33:04, 33:05, 34:02, 66:02, 68:03, 74:02, 74:03, 68:05; 01:02, 31:02, 34:01, 34:05 |
| HLA-A | A24 | 23:01, 24:02, 24:03, 24:09, 24:23, 24:35, 24:25, 24:07, 24:14, 24:32 |
| HLA-B | B07 | 07:02, 07:05, 35:01, 35:03, 42:01, 51:01, 51:02, 53:01, 54:01, 55:01, 55:02, 56:01, 67:01, 78:01; 07:04, 07:06, 35:08, 35:14, 35:31, 35:41, 35:43, 35:55, 39:10, 51:05, 51:08, 51:09, 53:06, 07:07, 35:02, 35:04, 35:05, 35:12, 35:17, 51:04, 51:13, 81:01, 15:29, 15:11, 35:20, 42:02 |
| HLA-B | B08 | 08:01, 08:09, 08:13, 08:04 |
| HLA-B | B27 | 14:02, 15:03, 15:09, 15:10, 15:18, 27:02, 27:03, 27:04, 27:05, 27:06, 27:07, 38:01, 39:01, 39:02, 39:09, 48:01, 73:01; 14:01, 38:09, 40:12, 48:02, 48:03, 15:23, 39:05, 39:06, 39:08, 39:24, 38:02, 35:16 |
| HLA-B | B44 | 18:01, 37:01, 40:01, 40:02, 40:06, 44:02, 44:03, 45:01; 18:03, 40:05, 40:11, 41:02, 44:04, 44:27, 50:01, 50:02; 18:14, 40:03, 40:42, 41:01, 44:05, 59:01; 13:04, 15:71, 18:04, 18:08, 40:08, 40:27, 44:10, 47:01, 47:02, 49:01, 51:07 |
| HLA-B | B58 | 15:16, 15:17, 57:01, 57:02, 58:01, 58:02, 57:03, 57:04 |
| HLA-B | B62 | 15:01, 15:02, 15:12, 15:13, 46:01, 52:01; 15:05, 15:15, 15:19, 15:25, 15:30, 52:04; 15:04, 15:07, 15:24, 15:35, 15:58, 15:73, 44:08; 13:01, 13:02 |
| HLA-C | C1 | 01:02, 03:02, 03:03, 03:04, 03:05, 03:06, 03:08, 03:09, 07:01, 07:02, 07:04, 07:06, 07:12, 07:15, 07:18, 07:21, 07:27, 08:01, 08:02, 08:03, 08:04, 08:10, 08:22, 12:02, 12:03, 12:13, 14:02, 14:03, 15:07, 16:01, 16:04 |
| HLA-C | C2 | 02:02, 02:03, 02:06, 02:10, 02:19, 03:07, 04:01, 04:03, 04:04, 04:05, 04:13, 05:01, 05:09, 05:13, 06:02, 06:07, 07:07, 12:05, 14:04, 15:02, 15:03, 15:04, 15:05, 15:06, 15:09, 15:11, 15:13, 16:02, 17:01, 17:03, 18:01, 18:02 |
| HLA Class II |  |  |
| HLA-DRB1 | DR1 | 15:01, 15:02, 15:03, 15:04, 16:01, 16:02 |
| HLA-DRB1 | DR3 | 03:01, 03:02, 03:05, 03:15, 07:01 |
| HLA-DRB1 | DR4 | 04:01, 04:03, 04:04, 04:05, 04:06, 04:07, 04:08, 04:10, 04:11, 14:01, 14:02, 14:04, 14:05, 14:06, 14:07, 14:24, 14:54 |
| HLA-DRB1 | DR5 | 01:01, 01:02, 01:03, 04:02, 08:01, 08:02, 08:03, 08:04, 08:06, 08:10, 08:11, 08:13, 10:01, 11:01, 11:02, 11:03, 11:04, 11:05, 11:15, 11:16, 12:01, 12:02, 13:01, 13:02, 13:03, 13:04, 13:05 |
| HLA-DRB1 | DR9 | 09:01 |

**Table S4. Concomitant medication use and drug interactions with clozapine**

| **Drug** | **Cases** | **Controls** |
| --- | --- | --- |
| Fluoxetine (*CYP2D6 and CYP2C19 inhibitor*) | 2 | 1 |
| Clobazam (*CYP2D6* inhibitor) | 0 | 0 |
| Escitalopram (*CYP2D6* inhibitor) | 0 | 4 |
| Sertraline (*CYP2D6* inhibitor) | 3 | 3 |
| Omeprazole (*CYP2C19* inhibitor) | 1 | 1 |
| Esomeprazole (*CYP2C19* inhibitor) | 0 | 4 |
| Ethinylestradiol (oral contraceptives) (*CYP2C19* inhibitor) | 1 | 1 |
| Rifampicin (*CYP3A4* inducer) | 0 | 0 |
| Macrolide antibiotics (*CYP3A4* inhibitor) | 0 | 0 |
| Azole antifungals (*CYP3A4* inhibitor) | 0 | 0 |
| Antivirals (*CYP3A4* inhibitor) | 0 | 0 |

**Figure S1a:** **Exonic variant gene association analysis - Broad** **Group**: 30 clozapine-induced myocarditis cases versus 54 clozapine-tolerant controls, adj MDS


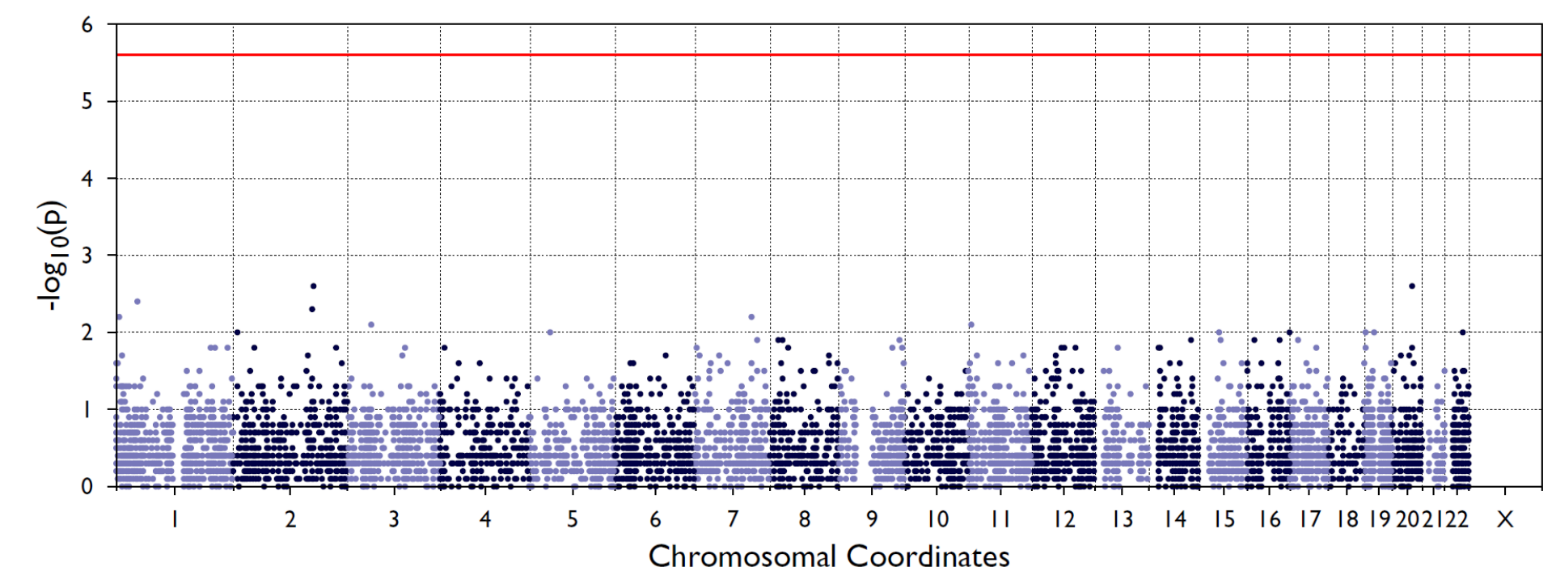


**Figure S1b:** **Exonic variant gene association analysis - Strict Group.** 30 clozapine-induced myocarditis cases versus 54 clozapine-tolerant controls, adj MDS


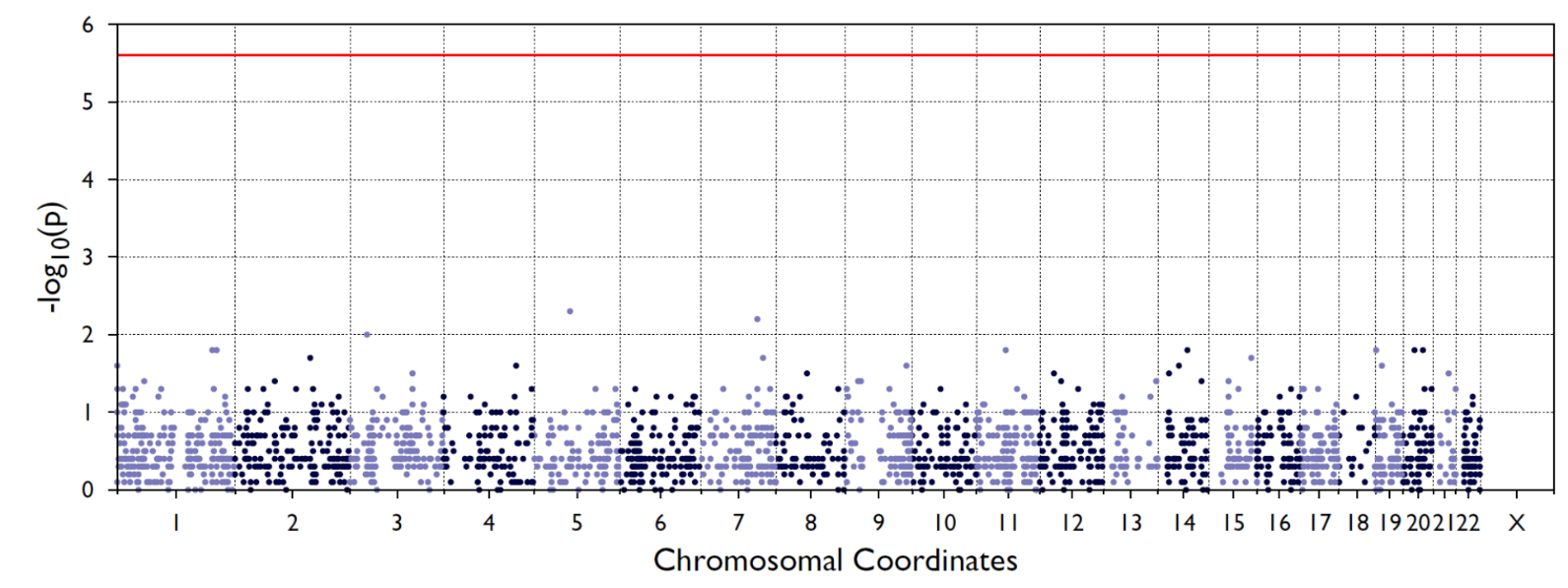


**Table S5: Long-read HLA-B calls**. Each HLA allele was analyzed separately. A filter of mapability of > 98 % is used to remove noisy type assignments. No further filters for read-depth were used. HLA-B allele B*07:02:01:01 was found to be the most differentially expressed allele where 18 out of 55 controls and 4 out of 33 cases expressed it. Overall no other alleles had significant differential expression.

| **Case** | **33** | **Control** | **55** |
| --- | --- | --- | --- |
| B*07:02:01:01 | 4 | B*07:02:01:01 | 18 |
| B*08:01:01:01 | 7 | B*08:01:01:01 | 8 |
| B*15:01:01:01 | 4 | B*15:01:01:01 | 3 |
| B*18:01:01:02 | 4 | B*18:01:01:02 | 7 |
| B*27:05:02:01 | 1 | B*27:05:02:01 | 2 |
| B*40:01:02:01 | 2 | B*40:01:02:01 | 1 |
| B*44:02:01:01 | 2 | B*44:02:01:01 | 1 |
| B*44:03:01:01 | 2 | B*44:03:01:01 | 2 |
| B*49:01:01 | 1 |  | |
| B*13:02:01:01 | 1 |  |  |
| B*15:21 | 1 |  |  |
| B*35:02:01:01 | 1 |  |  |
| B*35:08:01:01 | 1 |  |  |
| B*39:06:02:01 | 1 |  |  |
| B*39:24:01 | 1 |  |  |
|  | | B*51:01:01:01 | 1 |
|  |  | B*57:01:01 | 1 |
|  |  | B*07:05:01:01 | 1 |
|  |  | B*07:06:01 | 1 |
|  |  | B*15:01:01:04 | 1 |
|  |  | B*35:01:01:02 | 2 |
|  |  | B*35:03:01:01 | 1 |
|  |  | B*37:01:01 | 1 |
|  |  | B*38:01:01 | 1 |
|  |  | B*39:01:01:03 | 1 |
|  |  | B*15:02:01 | 1 |
|  |  | B*15:27:01 | 1 |

| **Table S6: HLA-allele discordance observed between long and short read sequencing.** | | | | | |  |
| --- | --- | --- | --- | --- | --- | --- |
| **ID** | **Phenotype** | **HLA allele** | **Long-read** | | **Short-read** | |
|  |  |  | **Allele_1** | **Allele_2** | **Allele_1** | **Allele_2** |
| MYC_026 | Control | HLA-A | 29:02:01:01 | ***29:02:01:01*** | 29:02:01 | ***29:05*** |
| MYC_090 | Case | HLA-B | 44:02:01:01 | ***44:02:01:01*** | 44:02:01 | ***44:03:01*** |
| MYC_186 | Case | HLA-C | ***16:01:01:01*** | 16:01:01:01 | ***03:04:01*** | 16:01:01 |
| MYC_314 | Control | HLA-C | ***04:01:01:05*** | 05:01:01:02 | ***04:09*** | 05:01:01 |
| MYC_332 | Case | HLA-C | ***06:02:01:01*** | 06:02:01:01 | ***03:04:01*** | 06:02:01 |
| Allele discordance are highlighted in bold and italics. | | | | | | |

| **Table S7: Univariate associations of clinical variables with clozapine-induced myocarditis.** | | |
| --- | --- | --- |
| **Clinical Variable** | **OR (95%CI)** | ***P*** |
| Sex | 1.562 (0.579 - 4.213) | 0.376 |
| Smoking | 1.33 (0.459 - 3.853) | 0.599 |
| Alcohol abuse | 1.737 (0.589 - 5.127) | 0.314 |
| Illicit drug use | 1.303 (0.464 - 3.656) | 0.615 |
| **Valproate use** | **3.099 (1.093 - 8.786)** | **0.029** |
| Age | 1.014 (0.976 - 1.053) | 0.474 |
| Age (per decade) | 1.143 (0.798 - 1.637) | 0.466 |
| BMI (kg/m^2^) | 1.07 (0.998 - 1.147) | 0.058 |
| Cumulative Clozapine Dose (mg) | 1.001 (0.999 - 1.002) | 0.221 |
| Cumulative Clozapine Dose (per 250mg) | 1.273 (0.854 - 1.896) | 0.181 |
| Significant univariate association with clozapine-induced myocarditis are highlighted in bold red font. | | |
| 29 clozapine-induced myocarditis cases were compared with 62 clozapine-tolerant controls. | | |
